# Leukotriene E_4_ is a full functional agonist for human cysteinyl leukotriene type 1 receptor-dependent gene expression

**DOI:** 10.1038/srep20461

**Published:** 2016-02-02

**Authors:** Holly R. Foster, Elisabeth Fuerst, William Branchett, Tak H. Lee, David J. Cousins, Grzegorz Woszczek

**Affiliations:** 1 Division of Asthma, Allergy and Lung Biology, King’s College London, UK; 2 MRC & Asthma UK Centre in Allergic Mechanisms of Asthma, UK; 3 NIHR Leicester Respiratory Biomedical Research Unit, Leicester Institute for Lung Health, Department of Infection, Immunity and Inflammation, University of Leicester, Leicester, UK

## Abstract

Leukotriene E_4_ (LTE_4_) the most stable of the cysteinyl leukotrienes (cysLTs) binds poorly to classical type 1 (CysLT_1_) and 2 (CysLT_2_) receptors although it induces potent responses in human airways *in vivo*, such as bronchoconstriction, airway hyperresponsiveness and inflammatory cell influx suggesting the presence of a novel receptor that preferentially responds to LTE_4_. To identify such a receptor two human mast cell lines, LAD2 and LUVA, were selected that differentially responded to LTE_4_ when analysed by intracellular signalling and gene expression. Comparative transcriptome analysis and recombinant gene overexpression experiments revealed CysLT_1_ as a receptor responsible for potent LTE_4_-induced response in LAD2 but not in LUVA cells, an observation confirmed further by gene knockdown and selective inhibitors. Lentiviral overexpression of CysLT_1_ in LUVA cells augmented intracellular calcium signalling induced by LTE_4_ but did not restore full agonist responses at the gene expression level. Our data support a model where both an increased expression of Gαq-coupled CysLT_1_, and sustained intracellular calcium mobilisation and extracellular signal-regulated kinase (Erk) activation, are required for LTE_4_-mediated regulation of gene expression in human cells. Our study shows for the first time that CysLT_1_ expression is critically important for responsiveness to LTE_4_ within a human cell system.

Cysteinyl leukotrienes (cysLTs) (LTC_4_, LTD_4_ and LTE_4_) play pivotal roles in cell proliferation, differentiation, migration and regulation of immune responses implicated in a wide variety of disorders, including asthma, allergy, atherosclerosis and cancer^1^. CysLTs are products of the 5-lipoxygenase (5-LO) pathway. 5-LO converts arachidonic acid to an unstable intermediate LTA_4_, which is then conjugated to reduced glutathione by leukotriene C_4_ synthase to form LTC_4_. After transport to the extracellular space LTC_4_ is converted to LTD_4_ and then to the terminal product LTE_4_, the most abundant cysLT in biological fluids. The biological actions of cysLTs are mediated by 2 currently identified G-protein coupled receptors (GPCR): cysLT type 1 (CysLT_1_) and 2 (CysLT_2_) receptors. They differ in binding affinities for different cysLTs. CysLT_1_ is recognized as a high-affinity receptor for LTD_4_, whereas CysLT_2_ binds LTC_4_ and LTD_4_ with similar affinity. LTE_4_, the most stable of the cysLTs, binds poorly to the classical CysLT_1_ and CysLT_2_ and is also much less potent than LTC_4_ and LTD_4_ in inducing cellular responses *in vitro*, showing a partial agonistic activity^2^,^3^,^4^,^5^. However *in vivo*, it is LTE_4_ that has shown to be the most potent cysLT in eliciting influx of eosinophils and basophils into bronchial mucosa of asthmatic subjects and in enhancing airway responsiveness to histamine and increasing vascular permeability, suggesting the existence of one or more leukotriene receptors that have not been identified to date^6^,^7^,^8^,^9^,^10^. The potential presence of such a receptor has been demonstrated in CysLT_1_/CysLT_2_ double knock-out mice^11^ but human data are lacking.

The observations that asthmatic airways respond with enhanced bronchoconstriction to inhaled cysLTs, especially to LTE_4_ in comparison with normal subjects^10^ and that infiltration of airways by mast cells is associated with disordered airway function in asthma^12^ suggest that mast cells could be a potential target cell type expressing a putative receptor preferentially responding to LTE_4_. In fact, the possible existence of such a novel, LTE_4_-activated receptor has been suggested in human mast cells^13^. In this study LTE_4_ has been shown to be the most potent of cysLTs in inducing cell proliferation and activation of gene expression in human primary mast cells and LAD2 human mast cell line. LTE_4_-mediated activities were resistant to knockdown of CysLT_1_ and CysLT_2_ but were dependent on PPAR-γ signalling. Another study has suggested that the P2Y12 receptor is required for LTE_4_-mediated responses^14^ but these observations have not been confirmed^15^.

In order to identify such a receptor responding to LTE_4_ we studied human mast cells and used transcriptome profiling by microarrays, recombinant GPCR overexpression models and methods analysing GPCR signalling. We characterize LTE_4_ as a fully functional agonist activating human CysLT_1_ and show for the first time that CysLT_1_ expression is critically important for responsiveness to LTE_4_ within a human cell system.

## Results

### LTE_4_ signals differently in LAD2 and LUVA cells

**Figure 1:**
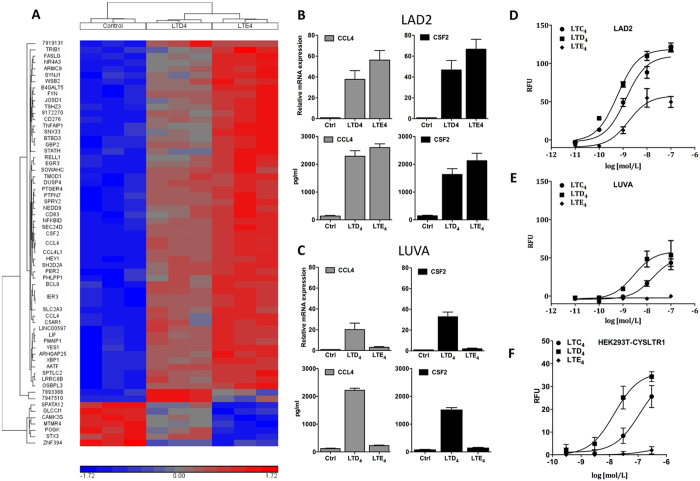
LTE_4_ is a potent agonist in LAD2 but not in LUVA cells. (**A**) LAD2 cells (n = 3) were stimulated with vehicle control, LTD_4_ and LTE_4_ and gene expression was analysed using microarrays. Hierarchical clustering of significantly (ANOVA, p < 0.05 LTD_4_ and LTE_4_ compared to control, False Discovery Rate = 0.1) regulated genes is presented as a heat map. (**B**) LAD2 and (**C**) LUVA cells were stimulated and CCL4 or CSF2 gene expression were measured at mRNA and protein levels. Data expressed as mean ± SEM from 3 separate experiments. (**D**) LAD2, (**E**) LUVA and (**F**) HEK293T transfected with CYSLTR1 cells were stimulated with indicated concentrations of LTC_4_, LTD_4_ and LTE_4_ and calcium mobilisation was measured. Data from 3 experiments run in triplicate, presented as mean ± SEM of baseline corrected peak intracellular calcium response. Relative fluorescence unit (RFU).

LTE_4_ has been shown to induce potent responses in LAD2 cells^13^ offering a model for identification of the elusive receptor responsible for LTE_4_ signalling. In order to compare responses between LTD_4_ and LTE_4_ microarray analysis of LAD2 cells stimulated with either vehicle control, LTD_4_ or LTE_4_ was carried out in the presence of L-cysteine (3 mmol/L) to inhibit dipeptidase enzyme responsible for converting LTD_4_ to LTE_4_^16^. Both leukotrienes significantly regulated expression of 64 genes including many chemokines, growth and transcription factors (Fig. 1A and supplementary Table 1). LTE_4_ was more potent in up and down regulation of gene expression than LTD_4_ for the majority of analysed genes, providing strong evidence for a robust LTE_4_ response in LAD2 cells. CCL4 and CSF2 were among the most upregulated genes in LAD2 cells and were selected for further analysis. qRT-PCR and ELISA analysis of LAD2 cells showed induction of CCL4 and CSF2 with LTE_4_ consistently matching or being the more potent of the 2 ligands (Fig. 1B). To verify whether this responsiveness to LTE_4_ is characteristic for other mast cells, another human mast cell line, LUVA, was analysed to compare responses to LTD_4_ and LTE_4_. Although in LUVA cells LTD_4_ regulated gene expression in a similarly potent way to LAD2 cells, LTE_4_ induced only very weak responses (Fig. 1C). As intracellular calcium mobilisation is a secondary messenger signalling cue for classical leukotriene receptors, cysLT induced calcium mobilisation was analysed in both cell lines. In LAD2 cells, all cysLTs induced a concentration-dependent calcium mobilisation (Fig. 1D), with LTD_4_ and LTC_4_ showing similar potency (LTC_4_ EC_50_−1.3 × 10^−9^ M, LTD_4_ EC_50_−0.58 × 10^−9 ^M) and LTE_4_ being the weakest of all 3 ligands but still inducing a robust response (LTE_4_ EC_50_−1.67 × 10^−9 ^M). In contrast, LTD_4_ was the most potent ligand in LUVA cells (EC_50_−2.8 × 10^−9 ^M) followed by LTC_4_ (EC_50_−1.7 × 10^−8 ^M), while LTE_4_ induced very weak response (EC_50_-not determined)(Fig. 1E). Similar potencies of cysLTs as in LUVA cells were detected in HEK293T cells transfected with human CYSLTR1 (Fig. 1F) (LTC_4_ EC_50_−1.12 × 10^−8 ^M, LTD_4_ EC_50_−0.9 × 10^−9 ^M; LTE_4_ EC_50_−8.32 × 10^−8 ^M). Therefore LAD2 and LUVA cells represent two human mast cell lines that respond differently to LTE_4_ stimulation.

### Comparison of GPCR gene expression profiles between LAD2 and LUVA cells

**Figure 2:**
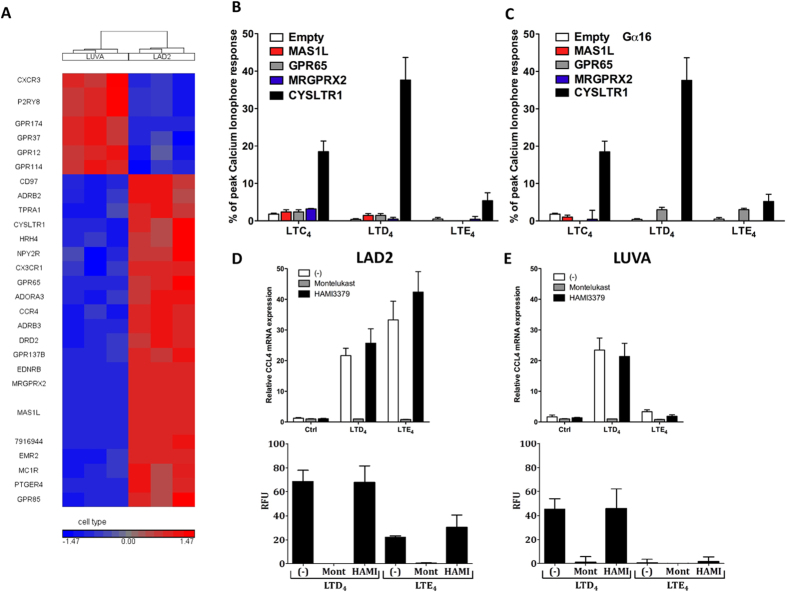
Comparison of GPCR gene expression profiles between LAD2 and LUVA cells. (**A**) Microarray gene expression was compared in LAD2 and LUVA cells (n = 3) and hierarchical clustering of differentially expressed GPCRs (ANOVA, p < 0.05, >2 fold difference) is presented as a heat map. Intracellular calcium mobilisation was analysed in HEK293T cells transiently transfected with the genes of interest (**B**) and co-transfected with Gα16 (**C**). Data expressed as percentage of peak calcium ionophore response, mean ± SEM from 3 experiments run in triplicate. LAD2 (**D**) and LUVA (**E**) cells were pre-treated with Montelukast (100 nmol/L) and HAMI3379 (1 μmol/L) for 10 minutes, stimulated with LTD_4_ and LTE_4_ (both 100 nmol/L) and CCL4 mRNA expression or calcium mobilisation was measured. Data expressed as a fold difference in comparison to vehicle control or as baseline corrected peak calcium response. Mean ± SEM from 3 separate experiments. Relative fluorescence unit (RFU).

A previous study^13^ has suggested that in LAD2 cells LTE_4_ signals through a novel, CysLT_3_ receptor, different from classical CysLT_1_ and CysLT_2_. As our observations in LAD2 and LUVA cells indicated that a potential LTE_4_ receptor should be differentially expressed in LAD2 and LUVA cells, gene expression was compared between LAD2 and LUVA cells using microarray in order to identify the putative gene. A list of significantly differentially expressed genes (ANOVA p < 0.05; >2 fold difference) was generated and GPCR genes were filtered using the IUPHAR GPCR database^17^. Among 27 GPCRs that differed significantly in expression between LAD2 and LUVA cells (Fig. 2A, supplementary Table 2), 10 GPCRs were considered orphan receptors (without known ligands)(GPR12, GPR37, GPR65, GPR85, GPR114, GPR137B, GPR174, MAS1L, MRGPRX2 and P2RY8). GPR65, MAS1L and MRGPRX2 were the most differentially expressed orphan GPCRs (9.9, 32.4 and 70.2 fold difference between LAD2 and LUVA cells, respectively). To ascertain whether cysLTs, and LTE_4_ in particular, could mediate signalling through any of these receptors, plasmids encoding GPR65, MAS1L and MRGPRX2 were transiently transfected into HEK293T cells and calcium mobilisation was analysed upon stimulation with cysLTs (Fig. 2B). CYSLTR1 gene was among differentially expressed GPCRs and was used as a positive control for all experiments. No specific calcium responses were observed in any of the transfectants apart from cells transfected with CYSLTR1, which showed the predicted pattern of response to cysLTs. As co-transfections of GPCRs and Gα_16_ have been reported previously to direct signal transduction to phospholipase C and calcium signalling^18^, target genes were co-expressed with human Gα_16_ and responses to cysLTs measured using calcium mobilisation in order to analyse potential alternative GPCR signalling pathway. Similarly, no response was observed in any of our overexpression models apart from CYSLTR1 transfected cells (Fig. 2C). Thus CysLT_1_ was the receptor that was differentially expressed in LAD2 and LUVA cells (4.3 fold difference) and responded to cysLTs.

### CysLT_1_ is required for LTE_4_ induced signalling in LAD2 cells

**Figure 3:**
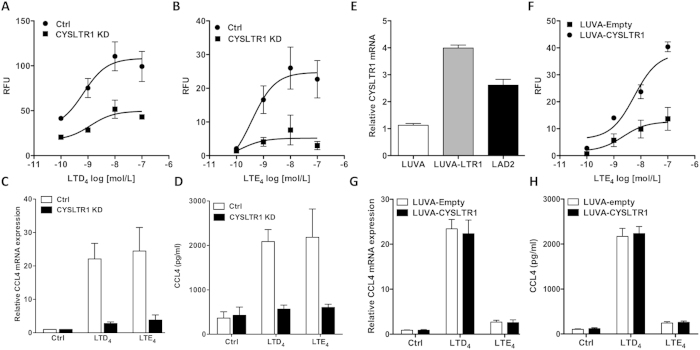
CysLT_1_ is required for LTE_4_ induced signalling in LAD2 cells. Calcium mobilisation responses to LTD_4_ (**A**) and LTE_4_ (**B**) in Empty control (Ctrl) and CYSLTR1 knocked down (CYSLTR1 KD) LAD2 cells. Baseline corrected peak calcium responses from 3 experiments run in triplicate presented as mean ± SEM. (**C**) Control and CYSLTR1 knocked down LAD2 cells were stimulated with vehicle control, LTD_4_ or LTE_4_ for 2 (mRNA) (**C**) or 6 hours (protein) (**D**) before analysis. Data expressed as fold difference in comparison to vehicle control for CCL4 mRNA and as CCL4 supernatant concentrations. Mean ± SEM from 3–5 experiments, relative fluorescence unit (RFU). (**E**) LUVA cells were stably transduced with empty (LUVA-empty) or CYSLTR1 overexpression (LUVA-CYSLTR1) vectors and relative CYSLTR1 mRNA expression was measured and compared to LAD2 cells. Mean ± SEM, n = 6. (**F**) Calcium mobilisation response to a range of LTE_4_ concentrations was evaluated in empty control and CYSLTR1 transduced LUVA cells. Mean ± SEM of baseline corrected peak calcium responses, n = 9. Control empty vector and CYSLTR1 transduced LUVA cells were stimulated as indicated before CCL4 mRNA (**G**) or protein (**H**) expression was measured. Mean ± SEM of 3 separate experiments.

To determine whether CysLT_1_ could be involved in LTE_4_ signal transduction, LAD2 and LUVA cells were pretreated with selective CysLT_1_ and CysLT_2_ antagonists, Montelukast and HAMI3379, respectively. Antagonists’ selectivity was previously verified in HEK293T cell transfection models (supplementary Figure 1). qRT-PCR analysis of CCL4 gene expression in LAD2 cells showed that both LTD_4_ and LTE_4_ induced responses were fully inhibited by Montelukast while HAMI3379 had no effect (Fig. 2D). In LUVA cells, LTD_4_ signalling was again fully inhibited by Montelukast but not by HAMI3379 (Fig. 2E). Analysis of calcium mobilisation in these cells showed a very similar picture, with Montelukast fully inhibiting LTE_4_ responses in LAD2 as well as LTD_4_ responses in LAD2 and LUVA cells while HAMI3379 had no effect (Fig. 2D,E). To verify whether the potent LTE_4_ induced, Montelukast sensitive, response in LAD2 cells was attributable specifically to CysLT_1_ signalling and not via another Montelukast sensitive receptor, stable CYSLTR1 receptor knockdown was generated in LAD2 cells using shRNA. Four shRNA targeting different regions of CYSLTR1 were transduced into separate LAD2 cell populations using lentiviral particles. qRT-PCR analysis of CYSLTR1 revealed shRNA “475” to significantly knock down CYSLTR1, without affecting CYSLTR2 mRNA expression (supplementary Figure 2). Knocking down of CYSLTR1 substantially inhibited intracellular calcium responses to LTD_4_ and LTE_4_ (Fig. 3A,B), confirming a functional decrease in CysLT_1_ expression. CCL4 and CSF2 mRNA and protein expression upon LTD_4_ and LTE_4_ stimulation were almost completely abrogated in CysLT_1_ knocked down LAD2 cells (Fig. 3C,D and supplementary Figure 3) identifying CysLT_1_ as a receptor responsible for LTE_4_ induced signalling in LAD2 cells.

### Overexpression of CysLT_1_ in LUVA cells does not determine LTE_4_ responses

Our GPCR expression profiles identified CYSLTR1 as more highly expressed in LAD2 than in LUVA cells. To test the hypothesis that the expression level of CYSLTR1 is relevant for mast cell responsiveness to LTE_4_, CYSLTR1 was stably overexpressed in LUVA cells using lentiviral transduction and positive clones were selected using puromycin. qRT-PCR confirmed a 3-fold increase in CYSLTR1 expression in the transduced population, a level similar to LAD2 cells (Fig. 3E). Functional CYSLTR1 overexpression was confirmed using calcium assay and showed potent concentration-dependent increase in LTE_4_ induced calcium responses (Fig. 3F), again similar to responses observed in LAD2 cells. Stimulation of LUVA cells overexpressing CysLT_1_ and control empty vector-transduced cells with either LTD_4_ or LTE_4_ revealed no significant differences in CCL4 mRNA or protein induction between both cell lines (Fig. 3G,H), showing that the expression level of CysLT_1_ does not solely determine LTE_4_ induced gene regulation, even though it allows for enhanced calcium mobilisation in response to LTE_4_.

### Comparison of CYSLTR1 gene sequence between LAD2 and LUVA cells

As genetic variations in the CYSLTR1 gene between LAD2 and LUVA cells could account for such differential responses to LTE_4_, promoter and coding regions of CYSLTR1 in both cell types were sequenced. DNA was extracted and CYSLTR1 promoter fragment containing 4 single nucleotide polymorphisms (SNPs)(rs321029, rs2637204, rs2806489, rs7066737) as well as the entire coding region were PCR amplified and sequenced. BLAST analysis of DNA sequences from LAD2 and LUVA cells revealed no differences between cell lines and showed that both cell lines share the same promoter homozygous haplotype, “CAAC” for 4 SNPs studied, respectively and homozygous T allele for rs320995 coding synonymous SNP. Human CYSLTR1 gene is localized to chromosome X thus lack of heterozygosity at the locus was consistent with the fact that both cell lines were derived from male donors.

### CysLT-activated CysLT_1_ signals through Gαq, calcium and Erk for gene regulation

**Figure 4:**
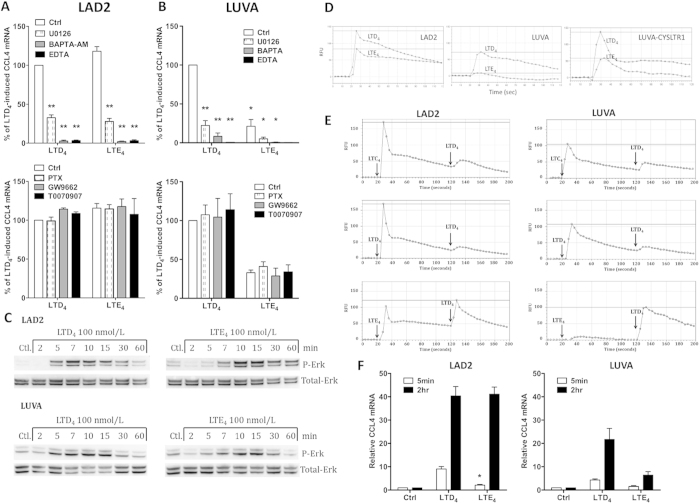
LTE_4_ induces sustained signalling in LAD2 cells. LAD2 (**A**) and LUVA (**B**) cells were pre-treated with selected inhibitors and stimulated with LTD_4_ or LTE_4_. Data from 3 separate experiments shown as % of LTD_4_-induced CCL4 mRNA expression (mean ± SEM), *p < 0.05, **p < 0.001, ANOVA with Bonferroni post test compared to LTD_4_ or LTE_4_. (**C**) LAD2 and LUVA cells were stimulated for time indicated with vehicle control (Ctrl), LTD_4_ or LTE_4_ (both 100 nmol/L) and phosphorylated Erk and total Erk expression measured using specific antibodies. Results from a representative experiment of 3 performed. Calcium mobilisation traces of LAD2, LUVA or LUVA-CYSLTR1 cells stimulated as indicated with LTD_4_ or LTE_4_ (100 nmol/L) once (**D**) or twice (**E**). Representative of 3 separate experiments, relative fluorescence unit (RFU). Black arrows indicate start of stimulation. (**F**) LAD2 and LUVA cells were exposed for either 5 minutes or 2 hours to vehicle control, LTD_4_ or LTE_4_ (both 100 nmol/L) and CCL4 mRNA measured by qRT-PCR after 2 hours incubation. Mean ± SEM data from 3 experiments shown as a fold change in comparison to controls. *p < 0.05, 2-way ANOVA comparison between 5 min LTD_4_ and LTE_4_ stimulations.

In order to compare CysLT_1_ mediated signalling in LAD2 and LUVA, both cell types were pre-incubated with several signalling pathway inhibitors and gene expression was measured in response to LTD_4_ and LTE_4_ (Fig. 4A,B). LTD_4_- and LTE_4_-induced CCL4 mRNA expression was potently inhibited by U0126 (MEK/Erk pathway inhibitor), intracellular (BAPTA-AM) and extracellular (EDTA) calcium chelators but was not modified by pertussis toxin or GW9662 and T0070907 (PPAR-γ inhibitors), suggesting that in both cell lines CysLT_1_ couples to Gαq, requires intracellular and extracellular calcium and Erk activation for regulation of gene expression.

### LTE_4_ activates prolonged signalling in LAD2 cells

To further analyse the agonistic activity of LTE_4_, time course experiments of Erk phosphorylation were conducted and analysed by Western blotting (Fig. 4C). In LAD2 cells stimulated with LTD_4_, Erk phosphorylation peaked at 7 minutes with a gradual decrease until 60 minutes. LTE_4_ induced a peak of Erk phosphorylation later but with a more sustained phosphorylation, still being detectable after 60 minutes. In LUVA cells the time point of highest Erk phosphorylation was similar to LAD2 cells but LTE_4_-induced Erk phosphorylation was shorter than in LAD2 cells. Thus sustained Erk phosphorylation induced by LTE_4_ in LAD2 but not in LUVA cells underlies an important difference in CysLT_1_-mediated responses between the cell lines.

We next compared calcium mobilisation kinetics in LAD2 cells; although LTD_4_ induced a higher peak response than LTE_4_, the intracellular calcium levels decreased at a higher rate after LTD_4_ stimulation while LTE_4_ induced a long lasting plateau phase (Fig. 4D). The sustained calcium signalling in response to LTE_4_ was not observed in LUVA cells or in LUVA cells overexpressing CysLT_1_ (Fig. 4D).

As GPCR signalling is regulated through receptor desensitization, cross desensitization experiments with cysLTs were performed (Fig. 4E). Prior stimulation with either LTC_4_ or LTD_4_ completely abrogated calcium response to LTD_4_ in LAD2 and LUVA cells, suggesting that both LTC_4_ and LTD_4_ can fully desensitize CysLT_1_ in both cell lines. However, prior stimulation with LTE_4_ caused only partial inhibition of the calcium response to LTD_4_ in LAD2 and LUVA cells, showing partial agonistic/desensitizing activity of LTE_4_ but no difference between the cell lines in LTE_4_ mediated signalling. The sustained increased level of calcium in LAD2 but not in LUVA cells after LTE_4_ stimulation was again the main difference observed between the cell lines in these experiments.

To analyse whether prolonged calcium/Erk signalling induced by LTE_4_ in LAD2 cells affects gene expression, CCL4 mRNA expression was analysed in LAD2 and LUVA cells after short (5 minutes) and long (2 hours) term exposure to LTD_4_ and LTE_4_, respectively. In LAD2 cells, 2 hour exposure to LTE_4_ and LTD_4_, caused similar upregulation of CCL4 mRNA expression (Fig. 4F). Stimulation of LAD2 cells with LTE_4_ for only 5 minutes failed to induce potent CCL4 expression with mRNA levels being significantly lower than that induced by LTD_4_. In LUVA cells, no difference could be observed between different exposure times.

## Discussion

This study identifies LTE_4_ as a fully functional agonist activating human CysLT_1_ for regulation of gene expression in LAD2 cells although only weak, partial agonism of LTE_4_ signalling could be detected in LUVA cells. Our data suggest that increased expression of CysLT_1_ and induction of prolonged intracellular signalling are required for LTE_4_ functional agonism. Ever since the elucidation and cloning of human CysLT_1_ and CysLT_2_, LTE_4_ has been considered as a final, non-active leukotriene metabolite due to its weak efficacy in recombinant systems and poor binding affinities compared to LTC_4_ and LTD_4_^6^. However, it was LTE_4_ that was shown to be the most potent cysLT in inducing inflammatory and contractile responses in asthmatic subjects. Our observation that LTE_4_ can induce full agonistic activity through CysLT_1_ could be of relevance for explaining this discrepancy between potent *in vivo* activity of LTE_4_ observed in asthmatic patients and weak *in vitro* potency for classical cysLT receptors. Early studies analysing the effects of cysLTs *in vivo* revealed a disproportionate augmentation in relative responses to LTE_4_ inhalation in asthmatic patients when compared to healthy individuals^10^. LTE_4_ responsiveness was increased more than 200 fold in asthmatics while responses to LTC_4_ and LTD_4_ were increased 6 and 9 fold respectively. More recent clinical studies suggest that CysLT_1_ is more highly expressed in asthmatic airways compared to healthy individuals^19^, with further increase observed in asthma exacerbations and in a sub-phenotype of asthma, patients with aspirin-exacerbated respiratory disease (AERD)^20^,^21^. This increased CysLT_1_ expression observed in AERD patients was significantly decreased following successful aspirin desensitisation, a procedure associated also with a significant reduction in sensitivity to inhaled LTE_4_^20^. We found similar disproportionate augmentation in LTE_4_-induced responses when comparing LAD2 and LUVA cells, a model of relatively high versus low CysLT_1_ expression. LTC_4_ and LTD_4_ were 2–3 times more potent at inducing calcium mobilisation in LAD2 cells compared to LUVA while LTE_4_ showed nearly 60-fold difference. Such potent responses to cysLTs, including LTE_4_ have been recently described in other human primary cells expressing high levels of CysLT_1_, T helper type 2 (Th2) lymphocytes^22^,^23^ and group 2 innate lymphoid cells (ILC2)^24^ supporting further our observation.

Prolonged intracellular signalling was identified as another potential contributing factor for the potency of LTE_4_ responses. The sustained increase in intracellular calcium and Erk phosphorylation upon LTE_4_ stimulation were observed in LAD2 but not in LUVA cells, suggesting that prolonged signalling could be critical for transcriptional regulation. LTE_4_, in contrast to LTC_4_ and LTD_4_, shows only partial activity and does not desensitise CysLT_1_ responses, a feature that can contribute to prolonged signalling in response to LTE_4_ in LAD2 cells. In fact, in experiments with short term exposure to agonists LTE_4_ showed only weak, partial agonist activity in comparison to LTD_4_, confirming important role of prolonged signalling in LTE_4_ induced responses. Overexpression of CysLT_1_ in LUVA did not restore sustained intracellular calcium and full agonism even though it increased peak calcium response to LTE_4_, suggesting that additional unidentified signalling molecules expressed in LAD2 cells but not in LUVA, are also required for full functional agonism of LTE_4_.

Mouse models provide strong evidence that CysLT_1_ and CysLT_2_ are not the only cysLT receptors as germline deletion did not diminish leukotriene-mediated inflammation^11^,^14^,^25^. Our data presented here provide an explanation for potent LTE_4_ activity observed in humans but do not rule out the possibility of another cysLT receptor. Our study shows for the first time that CysLT_1_ expression is critically important for responsiveness to LTE_4_ within a human cell system. This could potentially be relevant for human cell types other than mast cells and could thus have important implications for diagnostics and targeted treatment of specific phenotypes of asthma.

## Materials and Methods

### Reagents

Leukotrienes (LTC_4_, LTD_4_ and LTE_4_), Montelukast, MK-571, HAMI3379, U-0126, GW9662, T0070907 (all Cayman Chemical), EDTA (Ambion), BAPTA-AM, Pertussis Toxin, Calcium ionophore (A23187), (all Sigma-Aldrich) were obtained from the manufacturers.

### Cell Culture

HEK293T cells were cultured in DMEM medium supplemented with 2 mmol/L glutamine, 10% fetal bovine serum and Penicillin/Streptomycin (50 units/ml) (all Life Technologies) in a humidified 5% CO_2_ 37 ^o^C incubator. LAD2 cells (a kind gift from Dr. Arnold Kirshenbaum, NIAID, NIH, USA^26^) and LUVA cells (a kind gift from Dr. John Steinke, University of Virginia, USA^27^) were cultured in StemPro-34 medium supplemented with with L-glutamine (2 mmol/L), Pen/Strep (50 IU/ml) and with or without stem cell factor (SCF) (100 ng/ml) (all Life Technologies), respectively. Cells were hemidepleted weekly with fresh medium.

### Transient transfections

HEK293T cells cultured to above 60% confluence were transiently transfected as described previously^4^,^15^ with a mixture of Lipofectamine 2000 (Life Technologies) and the following plasmids as indicated: pcDNA3.1-empty, pcDNA3.1-human CYSLTR1, pcDNA3.1-human CYSLTR2, pcDNA3.1-human GPR65, pcDNA3.1-human GNA15 (Gα_16_) (all the Missouri S&T cDNA Resource Center, Rolla, Mo) and pCMV6-Kan/Neo- human MAS1L and human MRGPRX2 (Origene Technologies) in serum-free medium (Opti-MEM, Life Technologies) according to manufacturer’s protocol. After incubation the transfection medium was removed and HEK293T cells were cultured for 36 hours before calcium mobilisation was assayed in response to stimulation with calcium ionophore (1μmol/L), LTC_4_, LTD_4_ and LTE_4_ (all 100 nmol/L).

### Short hairpin RNA (shRNA) knockdown

For stable gene silencing shRNA constructs targeting different regions of human CYSLTR1 (clone ID: V3LHS_305475, V3LHS_305478, V2LHS_90946 and V2LHS_90947) were purchased from ThermoScientific and used to generate lentiviral particles with the lentiviral packaging system (psPAX2, pMD2.G and PEG-it™ precipitation)(System Biosciences) according to manufacturer’s protocol. LAD2 cells were transduced with viral particles for 24 hours and positive cells selected using Puromycin (2μg/ml)(Life Technologies). Efficiency of transduction was assessed by analysing GFP expression using flow cytometry.

### CYSLTR1 overexpression

CYSLTR1 gene was amplified from the pcDNA3.1-CYSLTR1 construct (UMR cDNA Resource Center) with primers containing restriction enzyme sites for NheI and BamHI (5′-AGGTGCTAGCATGGATGAAACAGGAAATT and 5′-GCGGGGATCCCTATACTTTACATATTTC) and cloned into lentiviral vector pCDH (System Biosciences) encoding GFP and puromycin resistance under the EF1 promoter and a multiple cloning site under the CMV promoter. Viral particles were generated using lentiviral packaging system (System Biosciences). LUVA cells were transduced, selected with puromycin (2μg/ml) and transduction efficiency was evaluated by GFP expression using flow cytometry.

### DNA sequencing

Total DNA was extracted using DNeasy Tissue kit (Qiagen) and fragments of CYSLTR1 gene were amplified using Platinum Taq Polymerase High Fidelity (Invitrogen) following manufacturer’s protocol and primers: CYSLTR1 promoter 5′-AACTGGAGACTTGCAGGTTGCG, 5′-AACATCAAAGTGCTGCCCCAGG; CYSLTR1 coding region 5′-TCAATGCCTCACTACTATTGCTTG, 5′-TTGGTTTGGACTGGAAATGGG and sequenced by Source Bioscience Sanger service using custom designed primers: CYSLTR1 promoter 5′-TAAGATGGGAAGCAGGGACG, 5′-GGCTTCAATCAGCACATACC; CYSLTR1 coding region 5′-ATACCAAGTGCTTTGAGCC, 5′-GCATTTGGCTCTTTGGTG and 5′-GTTTGATTGTCTTGTGGGG.

### Calcium mobilisation assay

Calcium mobilisation assays were conducted using FLIPR calcium 4 assay kit (Molecular Devices) as described previously^4^,^28^. Cells (1.5 × 10^5^/well) were plated into poly-L-lysine coated 96 well plates in RPMI 1640 supplemented with 10 mmol/L HEPES, incubated for 1 hour with FLIPR loading buffer prior to addition of ligand and fluorescent intensity was measured at 37 °C using a Flexstation 3 (Molecular Devices). Controls included medium control with ethanol for leukotriene stimulations. Results were analysed with SoftMax Pro Software (Molecular Devices).

### Real time PCR

LAD2 and LUVA cells were stimulated for 2 hours in the presence of L-cysteine (3 mmol/L) with LTD_4_ and LTE_4_ (both 100 nmol/L) and vehicle control. In some experiments as indicated cells were pretreated with U0126 (1μmol/L; 30 min), BAPTA-AM (30μmol/L; 30 min), EDTA (2.5 mmol/L; 5 min), pertussis toxin (PTX)(100 ng/ml; overnight), GW9662 (10μmol/L; 30 min) or T0070907 (1μmol/L; 30 min). Total cellular RNA was isolated using the miRNeasy mini kit (Qiagen), DNAase treated (Ambion) and reverse transcribed using RevertAid M-MuLV (Fermentas). Expression of mRNA encoding selected genes was measured using real time PCR on an ABI Prism 7900 Sequence Detection System (Applied Biosystems). Commercially available primer probe sets: 18S rRNA - 4319413E (Applied Biosystems) and individually designed assays using the Universal Probe Library (UPL) (Roche): CYSLTR1- probe 71, primers 5′-GGAGAGGGTCAAAGCAACAA, 5′-TGCAGAAGTCCGTGGTCATA; CYSLTR2- probe 21, primers 5′-TGATGTGACACTGCCGTTCT, 5′-TCATGGCTTCCTCAATAATGC; CCL4- probe 20, primers 5′-CAGCACAGACTTGCTTGCTT, 5′-CTTCCTCGCAACTTTGTGGT; CSF2- probe 1, primers 5′-GCCCTTGAGCTTGGTGAG, 5′-TCTCAGAAATGTTTGACCTCC were used. All primers/probes were tested for optimal efficiency of amplification. Relative gene expression was normalized to 18S rRNA. Data were analysed using SDS2.1 software (Applied Biosystems).

### Microarray Analysis

Total cellular RNA was isolated using the miRNeasy mini kit (Qiagen), DNase treated (Ambion), quality analysed on an Agilent 2100 Bioanalyzer (Agilent Technologies) and further processed with the Ambion WT Expression Kit (Applied Biosystems) according to the manufacturers’ instructions^29^. cRNA was fragmented, labelled, and hybridised to the Affymetrix Human Gene 1.0 ST Arrays using the Gene Chip WT Terminal Labeling and Hybridization Kit (Affymetrix). GeneChip fluidics station 450 (Affymetrix) was used for processing of the arrays and fluorescent signals were detected with the GeneChip scanner 3000. Images were analysed with the GeneChip operating software (Affymetrix). Further analysis was performed with the Partek Genomics Suite (Partek). RMA processing and quantile normalization was applied, and after Median Polish and gene level probeset summarization, differentially expressed genes were identified using ANOVA. Data were submitted to Gene Expression Omnibus database (accession number GSE75603).

### Western Blot Analysis

Total protein lysates were prepared using lysis buffer containing 1 mM protease inhibitor cocktail (Roche), 25 μg proteins loaded onto a 10% Bis-Tris NuPage gel (Invitrogen) and transferred onto a nitrocellulose membrane (Invitrogen). The membrane was incubated with primary antibodies against phospho-p44/42 MAPK and p44/p42 MAPK (Extracellular-signal-regulated kinase (ERK))(Cell Signaling) overnight at 4 °C, followed by secondary, horseradish peroxidase-conjugated antibody (goat anti rabbit IgG (Southern Biotech). Blots were developed using ECL plus Detection Reagent (GE Healthcare) and visualized on a Chemidoc MP System (BioRad). Data were analysed using Image Lab 4.1 software (BioRad).

### ELISA

LAD2 and LUVA cells were stimulated for 6 hours in the presence of L-cysteine (3 mmol/L) with LTD_4_ and LTE_4_ (both 100 nmol/L) and vehicle control. CCL4 and CSF2 concentrations were measured in supernatants using human CCL4 (MIP-1β) and CSF2 (GM-CSF) duo set kits (R&D Systems, UK) following manufacturer’s protocol.

### Statistical analysis

Data were analysed by means of one- or two- way ANOVA using GraphPad Prism software (GraphPad). Differences were considered significant at a p-value of less than 0.05.

## Additional Information

**How to cite this article**: Foster, H. R. *et al.* Leukotriene E_4_ is a full functional agonist for human cysteinyl leukotriene type 1 receptor-dependent gene expression. *Sci. Rep.* **6**, 20461; doi: 10.1038/srep20461 (2016).

## Supplementary Material

Supplementary Information
