# Supplementary material for: Leukotriene E4 is a full functional agonist for human cysteinyl leukotriene type 1 receptor-dependent gene expression
Source: Sci Rep. 2016 Feb 2;6:20461. doi: 10.1038/srep20461 (PMC4735867; doi:10.1038/srep20461)
Supplement: Supplementary Information [file srep20461-s1.pdf]

Online supplementary material

Title: Leukotriene E_4 is a full functional agonist for human cysteinyl leukotriene type 1 receptor-dependent gene expression

Authors: H. R. Foster[1,2‡], E. Fuerst[1,2‡], W. Branchett[1,2], T. H. Lee[1,2,†], D. J. Cousins[1,2,3], G. Woszczek[1,2*]

Table 1. List of genes (probes) significantly regulated by stimulation with LTD_4 or LTE_4 in comparison to vehicle control in LAD2 cells (ANOVA, p<0.05, False Discovery Rate = 0.1).

	ID	Gene_assignment	Gene Symbol	Fold-Change (Control vs. LTD4)	Fold-Change (Control vs. LTE4)
1	15179	AY766446 // CCL4 // chemokine (C-C motif) ligand 4 // 17q12 // 6351 /// AY766447 // CCL	CCL4	-8.74218	-16.7445
2	16413	AY766446 // CCL4 // chemokine (C-C motif) ligand 4 // 17q12 // 6351 /// AY766447 // CCL	CCL4	-8.74218	-16.7445
3	15178	AY766447 // CCL4L1 // chemokine (C-C motif) ligand 4-like 1 // 17q12 // 9560 /// AY7664	CCL4L1	-7.27318	-14.2327
4	15176	AY766446 // CCL4 // chemokine (C-C motif) ligand 4 // 17q12 // 6351 /// ENST00000250151	CCL4	-6.27419	-16.7221
5	29743	AK292464 // EGR3 // early growth response 3 // 8p23-p21 // 1960 /// ENST00000317216 //	EGR3	-5.55947	-12.5264
6	13615	AF216224 // LINC00597 // long intergenic non-protein coding RNA 597 // 15q23-q24 // 816	LINC0059 7	-4.36168	-5.56335
7	18086	AF385434 // NFKBID // nuclear factor of kappa light polypeptide gene enhancer in B-cell	NFKBID	-3.15495	-5.53942
8	25305	BC108724 // CSF2 // colony stimulating factor 2 (granulocyte-macrophage) // 5q31.1 // 1	CSF2	-3.0202	-4.6602
9	4812	BC030607 // LRRC8B // leucine rich repeat containing 8 family, member B // 1p22.2 // 23	LRRC8B	-3.01226	-4.05937
10	29944	AF254637 // HEY1 // hairy/enhancer-of-split related with YRPW motif 1 // 8q21 // 23462	HEY1	-3.00429	-4.01398
11	6246	ENST00000464839 // GBP2 // guanylate binding protein 2, interferon-inducible // 1p22.2	GBP2	-2.81828	-4.74415

12	25033	L28175 // PTGER4 // prostaglandin E receptor 4 (subtype EP4) // 5p13.1 // 5734 /// ENST	PTGER4	-2.63092	-3.43541
13	27122	AF039067 // IER3 // immediate early response 3 // 6p21.3 // 8870 /// BC000844 // IER3 /	IER3	-2.61982	-3.43259
14	33032	AF039067 // IER3 // immediate early response 3 // 6p21.3 // 8870 /// BC000844 // IER3 /	IER3	-2.61982	-3.43259
15	29778	U21108 // DUSP4 // dual specificity phosphatase 4 // 8p12-p11 // 1846 /// ENST000002401	DUSP4	-2.61512	-3.12733
16	16610	D90070 // PMAIP1 // phorbol-12-myristate-13-acetate-induced protein 1 // 18q21.32 // 53	PMAIP1	-2.58693	-3.18639
17	32904	AF039067 // IER3 // immediate early response 3 // 6p21.3 // 8870 /// AF083421 // IER3 /	IER3	-2.51551	-3.31687
18	11755	BX648582 // SPRY2 // sprouty homolog 2 (Drosophila) // 13q31.1 // 10253 /// ENST0000037	SPRY2	-2.44659	-3.30198
19	23985	BC067219 // STATH // statherin // 4q13.3 // 6779 /// BX649104 // STATH // statherin //	STATH	-2.43164	-6.5496
20	30526	D78579 // NR4A3 // nuclear receptor subfamily 4, group A, member 3 // 9q22 // 8013 ///	NR4A3	-2.34479	-4.88248
21	18963	BC036652 // SOWAHC // sosondowah ankyrin repeat domain family member C // 2q13 // 65124	SOWAHC	-2.27147	-2.825
22	9302	---		-2.20735	-1.41984
23	6621	AF097744 // SH2D2A // SH2 domain containing 2A // 1q21 // 9047 /// ENST00000368199 // S	SH2D2A	-2.09425	-2.62985
24	26995	AK292682 // NEDD9 // neural precursor cell expressed, developmentally down-regulated 9	NEDD9	-2.01024	-2.61269
25	17456	BC008982 // C5AR1 // complement component 5a receptor 1 // 19q13.3-q13.4 // 728 /// ENS	C5AR1	-1.99449	-2.53677
26	29551	BC063292 // TRIB1 // tribbles homolog 1 (Drosophila) //	TRIB1	-1.95595	-4.18346

		8q24.13 // 10221 /// ENST000003			
27	22041	BC069540 // LIF // leukemia inhibitory factor // 22q12.2 // 3976 /// ENST00000249075 //	LIF	-1.89923	-2.26037
28	16663	ENST00000314574 // YES1 // v-yes-1 Yamaguchi sarcoma viral oncogene homolog 1 // 18p11.	YES1	-1.80077	-2.27664
29	6842	BC001746 // PTPN7 // protein tyrosine phosphatase, non-receptor type 7 // 1q32.1 // 577	PTPN7	-1.79981	-2.23885
30	6402	---		-1.78997	-1.68385
31	26261	BC030830 // CD83 // CD83 molecule // 6p23 // 9308 /// ENST00000379153 // CD83 // CD83 m	CD83	-1.74841	-2.42664
32	891	---		-1.74642	-1.26037
33	20462	AK294876 // PER2 // period homolog 2 (Drosophila) // 2q37.3 // 8864 /// ENST00000254657	PER2	-1.70673	-1.98817
34	32175	---		-1.6855	-2.31655
35	22028	BC012841 // XBP1 // X-box binding protein 1 // 22q12.1|22q12 // 7494 /// ENST0000021603	XBP1	-1.68466	-2.0767
36	24733	AF130464 // SEC24D // SEC24 family, member D (S. cerevisiae) // 4q26 // 9871 /// AK2917	SEC24D	-1.57894	-1.96342
37	20554	AK299281 // BTBD3 // BTB (POZ) domain containing 3 // 20p12.2 // 22903 /// ENST00000254	BTBD3	-1.55008	-2.05834
38	30513	BC002660 // TMOD1 // tropomodulin 1 // 9q22.3 // 7111 /// ENST00000259365 // TMOD1 // t	TMOD1	-1.52537	-1.66137
39	28503	ENST00000313367 // OSBPL3 // oxysterol binding protein-like 3 // 7p15 // 26031 /// ENST	OSBPL3	-1.5228	-1.80363
40	22125	BC015026 // JOSD1 // Josephin domain containing 1 // 22q13.1 // 9929 /// ENST0000021603	JOSD1	-1.46992	-1.92819
41	13100	AK291721 // CD276 // CD276 molecule // 15q23-q24 // 80381 /// ENST00000318443 // CD276	CD276	-1.45467	-1.77488
42	27502	M14333 // FYN // FYN oncogene related to SRC, FGR, YES // 6q21 // 2534 ///	FYN	-1.44559	-1.68454

		ENST00000368			
43	21486	AF009039 // SYNJ1 // synaptojanin 1 // 21q22.2 // 8867 /// ENST00000382499 // SYNJ1 //	SYNJ1	-1.43387	-1.92248
44	13125	EF653821 // SNX33 // sorting nexin 33 // 15q24.2 // 257364 /// ENST00000308527 // SNX33	SNX33	-1.40268	-1.90385
45	24452	BC039540 // RELL1 // RELT-like 1 // 4p14 // 768211 /// ENST00000314117 // RELL1 // RELT	RELL1	-1.38026	-1.68682
46	15101	AK300584 // TNFAIP1 // tumor necrosis factor, alpha-induced protein 1 (endothelial) //	TNFAIP1	-1.3767	-1.78605
47	18746	AK290396 // ARHGAP25 // Rho GTPase activating protein 25 // 2p13.3 // 9938 /// AK297056	ARHGAP2 5	-1.34839	-1.54089
48	21157	AB004550 // B4GALT5 // UDP-Gal:betaGlcNAc beta 1,4- galactosyltransferase, polypeptide	B4GALT5	-1.3373	-1.59854
49	10655	AB209607 // SLC2A3 // solute carrier family 2 (facilitated glucose transporter), member	SLC2A3	-1.33586	-1.4728
50	18051	AK291466 // TSHZ3 // teashirt zinc finger homeobox 3 // 19q12 // 57616 /// ENST00000240	TSHZ3	-1.33225	-1.66953
51	5298	D38122 // FASLG // Fas ligand (TNF superfamily, member 6) // 1q23 // 356 /// ENST000003	FASLG	-1.3298	-1.73157
52	12611	BC005123 // SPTLC2 // serine palmitoyltransferase, long chain base subunit 2 // 14q24.3	SPTLC2	-1.31585	-1.41128
53	16621	BC014927 // PHLPP1 // PH domain and leucine rich repeat protein phosphatase 1 // 18q21.	PHLPP1	-1.29498	-1.45803
54	5010	BC116451 // BCL9 // B-cell CLL/lymphoma 9 // 1q21 // 607 /// ENST00000234739 // BCL9 //	BCL9	-1.28676	-1.36759
55	19447	AB058771 // ARMC9 // armadillo repeat containing 9 // 2q37.1 // 80210 /// AY219922 // A	ARMC9	-1.2482	-1.4451
56	11220	AF163324 // WSB2 // WD repeat and SOCS box containing 2 // 12q24.23 // 55884 /// AF2291	WSB2	-1.23277	-1.37082

57	15187	BC000591 // AATF // apoptosis antagonizing transcription factor // 17q12 // 26574 /// E	AATF	-1.13876	-1.16356
58	8552	AK290251 // STX3 // syntaxin 3 // 11q12.1 // 6809 /// AK297419 // STX3 // syntaxin 3 //	STX3	1.02997	1.14411
59	7942	BC034044 // CAMK2G // calcium/calmodulin-dependent protein kinase II gamma // 10q22 //	CAMK2G	1.0915	1.23204
60	5262	AB040946 // POGK // pogo transposable element with KRAB domain // 1q24.1 // 57645 /// E	POGK	1.12278	1.38229
61	16176	BC035609 // MTMR4 // myotubularin related protein 4 // 17q22-q23 // 9110 /// ENST000003	MTMR4	1.2065	1.43276
62	22512	AY221117 // SPATA12 // spermatogenesis associated 12 // 3p14.3 // 353324 /// ENST000003	SPATA12	1.23352	1.5721
63	27750	ENST00000223145 // GLCCI1 // glucocorticoid induced transcript 1 // 7p21.3 // 113263 //	GLCCI1	1.2636	1.50484
64	28820	AY642122 // ZNF394 // zinc finger protein 394 // 7q22.1 // 84124 /// BC017051 // ZNF394	ZNF394	1.3302	1.43708

Table 2. Differentially expressed GPCRs in LAD2 cells compared to LUVA cells (ANOVA, p<0.05, > than 2 fold difference). Orphan receptors selected for analysis are highlighted.

Gene	Description	Orphan?	Fold difference LAD2 vs. LUVA
ADORA3	Adenosine A3 receptor	No	2.27
ADRB2	Adrenergic β2 receptor	No	2.39
ADRB3	Adrenergic β3 receptor	No	6.52
CCR4	Chemokine receptor	No	4.16
CD97	Adhesion class receptor	No	2.87
CX3CR1	Chemokine receptor	No	4.76
CXCR3	Chemokine receptor	No	-3.60
CYSLTR1	Cysteinyl leukotriene receptor 1	No	4.32
DRD2	Dopamine receptor D2	No	4.90
EDNRB	Endothelin receptor type B	No	6.83
EMR2	Adhesion class receptor	No	4.50
GPR12	G-protein coupled receptor 12	Yes	-2.41
GPR37	G-protein coupled receptor 37	Yes	-2.05
**GPR65**	**G-protein coupled receptor 65**	**Yes**	**9.93**
GPR85	G-protein coupled receptor 85	Yes	2.94
GPR114	Adhesion class receptor	Yes	-2.14
GPR137B	G-protein coupled receptor 137B	Yes	4.12
GPR174	G-protein coupled receptor 174	Yes	-8.04
HRH4	Histamine H4 receptor	No	5.06
**MAS1L**	**MAS1 proto-oncogene like receptor**	**Yes**	**32.41**
MC1R	Melanocortin 1 receptor	No	2.02
**MRGPRX2**	**MAS-related GPR, member X2**	**Yes**	**70.23**
NPY2R	Neuropeptide Y2 receptor	No	2.30
P2RY8	Purinergic receptor P2Y, 8	Yes	-6.34
7916944	Prostaglandin E receptor 3	No	11.40
PTGER4	Prostaglandin E receptor 4	No	3.96
TPRA1	Transmembrane protein, adipocyte associated 1	No	2.52

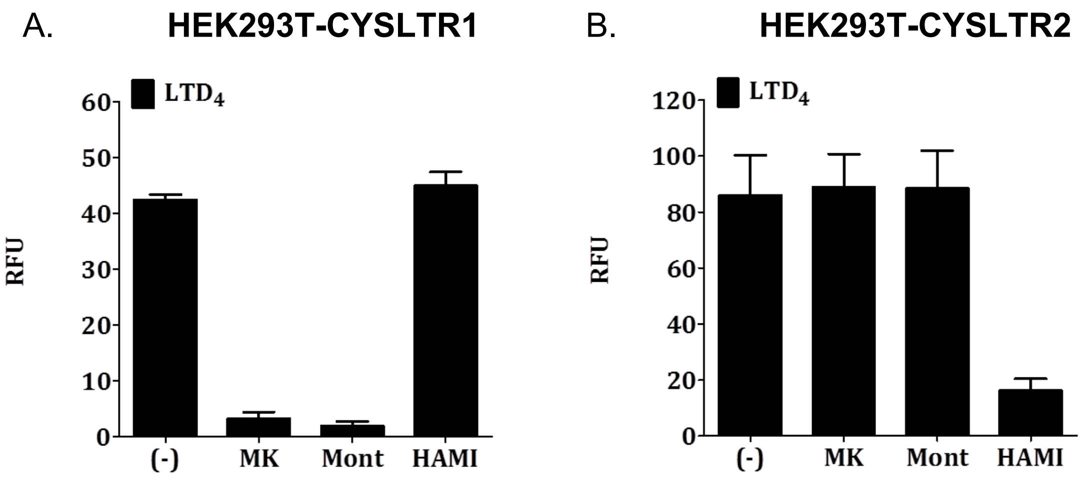

Supplementary Figure 1. The effect of inhibitors in HEK293T-CYSLTR1 and HEK293T-CYSLTR2 overexpression models. HEK293T cells were transiently transfected with human CYSLTR1 (A) or CYSLTR2 (B), preincubated with CYSLTR1 inhibitors (MK-571 (1 μmol/L), Montelukast (100 nmol/L)) or CYSLTR2 inhibitor (HAMI3379 (1 μmol/L)) before stimulation with LTD_4 (100 nmol/L). Baseline corrected peak calcium mobilisation is presented as mean ± SEM from 3 experiments run in triplicate. Relative fluorescence units (RFU).

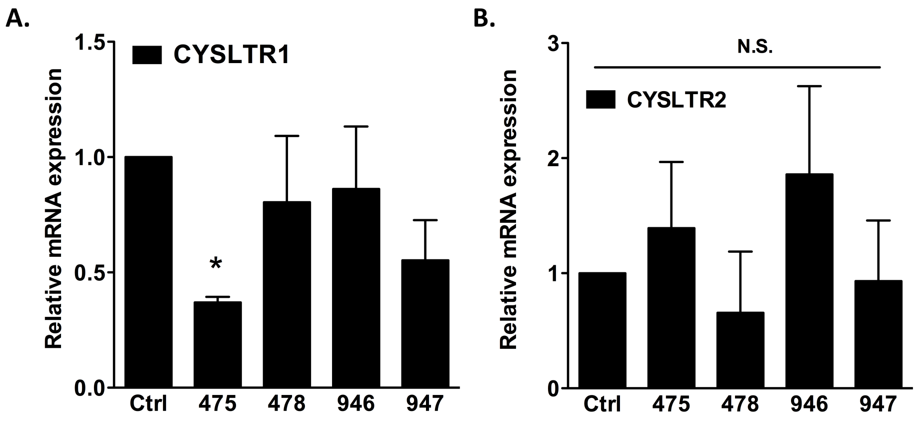

Supplementary Figure 2. Four different shRNAs (475, 478, 946, 947) targeting CYSLTR1 were stably transduced into LAD2 cells and expression of CYSLTR1 (A) and CYSLTR2 (B) mRNA analysed using qRT-PCR for verification of successful gene knockdown. Mean ± SEM of 3 experiments. * $p < 0.05$, One way ANOVA with Bonferroni post test in comparison to control.

A B

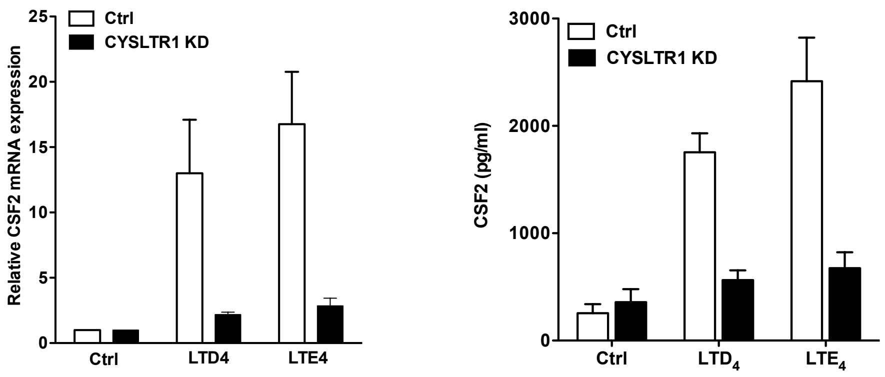

Supplementary Figure 3. $CysLT_1$ is required for LTE_4 induced CSF2 expression. Control and CYSLTR1 knocked down LAD2 cells were stimulated with vehicle control, LTD_4 or LTE_4 for 2 (mRNA) (A) or 6 hours (protein) (B) before analysis. Data expressed as fold difference in comparison to vehicle control for CSF2 mRNA and as CSF2 supernatant concentrations. Mean ± SEM from 3 experiments.
